# Connecting mothers to care: Effectiveness and scale-up of an mHealth program in Timor-Leste

**DOI:** 10.7189/jogh.09.020428

**Published:** 2019-12

**Authors:** Susan Thompson, Mary Anne Mercer, Marisa Hofstee, Bert Stover, Paul Vasconcelos, Sarah Meyanathan

**Affiliations:** 1 University of Washington, Seattle, Washington, USA; 2 Health Alliance International, Dili, Timor-Leste

## Abstract

**Background:**

Health Alliance International (HAI) with the Ministry of Health (MoH) of Timor-Leste and Catalpa International implemented a mobile phone-based mHealth program in 2013 known as *Liga Inan* (“Connecting Mothers”). Liga Inan was designed as a sustainable and scalable effort that would support MoH efforts to improve maternal and newborn health care-seeking and home practices. Key aims were to use mobile phone technology to improve communication between pregnant women and their MoH health providers and to increase optimal maternal health behaviors. MoH health staff registered pregnant women into Liga Inan at their first antenatal care (ANC) visit and followed them through pregnancy, delivery and six months postpartum. A web-based platform sent text messages twice weekly to promote safe pregnancy/delivery and facilitated phone communication between pregnant women and their MoH care providers.

**Methods:**

For the program’s final evaluation, baseline (2012) and final (2015) surveys interviewed women in one intervention district and one adjacent control district who had given birth in the preceding two years. Primary outcomes were receiving four or more ANC visits, using skilled birth attendants, delivery in health facilities, and timely postnatal care.

**Results:**

Multivariate analysis compared endline maternal health behaviors for women in the intervention district compared to baseline and to women in the control district. Controlling for other factors, women in the intervention district had nearly twice the odds of having a skilled birth attendant and a facility delivery, nearly five times the odds of receiving a postpartum care visit within two days of delivery, and over five times the odds of having their newborn’s health checked within two days of birth. There was no significant association between Liga Inan exposure and receipt of four or more ANC visits.

**Conclusions:**

Liga Inan was associated with substantial increases in MoH health provider-assisted and facility-based births and timely postnatal care in Timor-Leste. These positive results led the MoH to incorporate Liga Inan into the national maternal and child health program. To date the program has expanded to cover all 13 districts in the country, with gradual assumption of management and financial responsibility by the MoH under way.

According to the United Nations, there were 289 000 maternal deaths globally in 2013, equivalent to 800 women dying every day [1]. These deaths occur disproportionately in sub-Saharan Africa, South Asia, East Asia, and the Pacific, with the highest proportion of women dying during labor, delivery, and the immediate postpartum period [2]. Death from childbearing-related causes is clearly a major public health problem in low and middle-income countries.

In 2000, world leaders from 189 countries made history by adopting the UN Millennium Declaration, committing their nations to achieve eight Millennium Development Goals (MDGs). The fifth MDG focused on maternal health, aiming to reduce maternal mortality by 75% globally by 2015 [1]. The use of a skilled birth attendant (SBA) is a key means to reduce high maternal mortality, but it is estimated that every year 27% of women globally experience barriers to delivering with a doctor, nurse or midwife, resulting in a major obstacle to further progress [3-5].

East Timor, now Timor-Leste, voted for independence from Indonesia in 1999, and the new government declared universal free access to health care, including maternity services. However, the country has faced numerous challenges to improving the population’s health. Maternal and newborn mortality are major problems: the 2010 Demographic and Health Survey (DHS) reported the maternal mortality ratio (MMR) to be 557 per 100 000 live births, with neonatal mortality of 22/1000 live births [6]. Maternal morbidity and mortality continue to be exacerbated by low use of services and limited community understanding of optimal care-seeking behaviors. Even with the recommended four antenatal care (ANC) visits, adequate time for provider-client communication that would help assure a healthy pregnancy and delivery is limited. Additionally, significant socio-economic and geographic disparities limit access to maternal services. For example, in the 2010 DHS only 21% of rural Timorese women delivered with a skilled birth attendant compared to 59% of women living in an urban setting [6]. Access to delivery facilities is limited by gaps in both communication with and transportation to health facilities.

The potential to harness new technologies such as mobile phones to reduce disparities in access and use of maternal care and improve maternal and newborn outcomes is of growing interest. Like many other countries, Timor-Leste is skipping the fixed-line telephone infrastructure, moving directly into mobile phone technology. In 2003, only 2% of Timorese households reported owning a mobile phone [7]. By 2010, ownership had increased nationally to 61%, with a 600% increase in ownership since 2006 [8]. By 2015, 66% of all Timorese women and 77% of men owned a mobile phone [9].

Given such rapid growth in the use of mobile phones, their use to support health programs (known as mHealth) in low resource settings is also proliferating [10]. Numerous meta-analyses have reported the success of focused mHealth programs in increasing maternal and newborn health services utilization, although most also identify the need for more high quality studies documenting effectiveness [11-15]. A major gap in the research has been evidence for the sustainability of programs, including scale-up of small projects to the regional or national levels [16].

This article describes implementation research to evaluate the effectiveness of an mHealth program that was integrated by design into Ministry of Health (MoH) services, with the goal of increasing the likelihood of sustainability if proven effective. The program aimed to increase utilization of ANC, SBA, facility delivery, and timely postpartum care in Timor-Leste government facilities [17]. In an effort to respond to the mERA criteria for reporting on mHealth programs, we have included information about the content, context, and technical features of the Liga Inan intervention [18].

## The Liga Inan Mobile Phone Intervention

The MoH of Timor-Leste aims to assure that all pregnant women receive timely and appropriate care before, during, and after childbirth. Health Alliance International (HAI), a non-profit, non-governmental organization, has supported the country in that effort since independence. HAI and Catalpa International, a non-profit software design and development organization, launched the country’s first mHealth program in 2013. *Liga Inan*, or “Connecting Mothers” in the local Tetun language, uses mobile phone technology to support MoH efforts to improve maternal and newborn home care and care-seeking practices. It sends pregnant women health information via text messages and facilitates voice communication between women and health providers. The program continues to send child health messages for six months after delivery. The simple mobile technology on the ground is supported by a web-based application which is hosted online and connected to a GSM gateway (a system that enables the sending and receiving of Short Message Service). The service is accessed over the internet and allows tailoring of scheduled outgoing weekly messages, program performance monitoring and viewing registrations.

At the time of implementation of Liga Inan there were no other mHealth programs operating in Timor-Leste and no national mHealth strategy. However, at the start of the Liga Inan program, the majority of women living in the intervention district owned a mobile phone and 95% reported they had cell phone signal at home or within a five minute walk of home. There have been significant improvements in the electrical grid since Timor-Leste gained independence resulting in largely reliable electricity in the country. Other enabling factors included high levels of reading literacy among women (68%) and among women who owned a mobile phone, 99% reported they regularly used the text message function.

MoH staff (midwives, doctors, and clinic managers) in the intervention district of Manufahi were trained on the key functions of Liga Inan and registered pregnant women into the program at their first ANC visit. Participating facilities were provided an Android smart phone and phone credit worth US$ 10 per month. After registration, each pregnant woman received twice-weekly text messages with health information and reminders to seek care. The messages were timed to the gestational stage of the woman’s pregnancy and were sent every Monday and Thursday. To facilitate two-way communication, health staff could send broadcast text messages with informational announcements to all pregnant women registered in their area, and were prompted by Liga Inan to call each pregnant woman in their catchment area three weeks before her due date to check on the family’s birth plan. Women with questions or emergencies could use the service to request assistance from health staff.

The primary aim of this research was to determine whether pregnant women’s residence in Manufahi District during the period of the intervention was associated with differences in their use of maternal and newborn health services compared to before the intervention and with a control district. We also hoped to demonstrate that it was feasible to integrate the design of an mHealth program within the MoH system, augmenting the possibility of scalability and sustainability. This article is based on research conducted by HAI for the program’s final evaluation. Later, the MoH also elected to carry out their own independent evaluation, employing both a survey and additional evaluation methods [17].

## METHODS

### Study design and setting

Cross-sectional study data included baseline (n = 581) and final (n = 576) household surveys in two largely rural, adjacent districts: Manufahi, where Liga Inan was introduced in 2013, and the control district Ainaro, where the program was not implemented. The final survey was conducted after Liga Inan had been operational in all four sub-districts of Manufahi District for at least two years.

The survey employed cluster sampling within each district, using population data from the Timor-Leste 2010 Census. Sub-districts were stratified into eight enumeration areas, with nine women randomly selected from each enumeration area to be interviewed. Starting households in each hamlet were randomly selected for participation, with further households systematically selected from this geographic point. Sample size was determined using calculations for community clustered trials using a post-test only design [19], knowing that this would give us conservative sample size estimates, and that the pre-intervention measurements would increase power.

### Participants

For both surveys, women aged 15-49 years old with a child up to 24 months of age were eligible to participate. If two women fit that description within one household, we interviewed the mother of the youngest child. Each participant gave informed consent and refusals were rare.

### Data sources

The survey questionnaire was modeled on USAID’s Knowledge, Practices, and Coverage (KPC) Rapid CATCH (Core Assessment Tool on Child Health) Survey, a standardized questionnaire used worldwide [20], combined with questions from the 2010 Timor-Leste DHS [6]. It included additional questions on mobile phone ownership and use, and the final survey had further questions related to the women’s experience with Liga Inan. Both questionnaires were field tested and translated into the local Tetun language. The baseline interviews were conducted in February and March 2012 and final interviews in September and October 2015. All interviews were conducted in Tetun.

### Statistical analysis

Bivariate results are presented as frequencies for each district, using chi square tests to determine statistical significance of differences between districts. Multivariate results were weighted to account for differences in population size between the sub-districts and between Manufahi and Ainaro Districts. Multivariate analysis assessed the extent to which potential exposure to the mHealth intervention predicted improved maternal care seeking behaviors compared to the pre-intervention time period and to those outcomes in the control district, Ainaro. Data were entered using Epi Info^TM^ 7.0, (CDC, Atlanta, GA, USA) and analysis was performed using Stata 14 (StataCorp, College Station, TX, USA) statistical software.

We used multilevel mixed-effects logistic regression models separately for each behavior outcome response, and adjusted for sub-district clustering of measurements by using random effects at the sub-district level in all models. Analysis was by intention to treat. The predictor of interest was the Liga Inan intervention delivered at the district level, and the outcomes were women’s utilization of key maternal health services. The control variables age, years of education, travel time to a health facility, and wealth index were included because there were significant district-level differences for those characteristics in either the baseline or final survey. The wealth index was constructed from five survey questions, allowing one point for each response that indicated higher wealth. These included home electricity, motorcycle or car, radio or TV, iron roof, and concrete or wooden floor. For postpartum and postnatal care, we also included skilled birth attendance as a control variable. Data are presented as odds ratios with 95% confidence intervals. A *P*-value of <0.05 for both bivariate and multivariate analyses was considered statistically significant.

HAI received Internal Review Board (IRB) approval for baseline and final KPC surveys from the University of Washington Human Subjects Division and from the Timor-Leste National Institute of Health.

## RESULTS

### Demographics of the study population

**Table 1** shows the characteristics of the study sample as observed at two cross-sectional assessments. Demographic differences between the control and intervention districts were inconsistent: in the baseline survey there were significant differences only for age and travel time to a health facility, while in the final survey differences were seen only for educational level and the wealth index.

**Table 1:** District-level background characteristics in the baseline survey compared to final survey and significance of differences between groups

Characteristics	Baseline survey			*P*-value	Final survey		*P*-value
**Intervention, 2012**		**Control, 2012**		**Intervention, 2015**	**Control, 2015**	
**Age (years)**				0.002			0.108
15-24	115 (39%)	77 (27%)			103 (36%)	92 (32%)	
25-29	82 (28%)	87 (30%)			89 (31%)	80 (28%)	
30-34	40 (14%)	67 (23%)			62 (22%)	60 (21%)	
35+	56 (19%)	57 (20%)			34 (12%)	55 (19%)	
**Education (years):**				0.911			0.010
Primary (0-8)	172 (59%)	172 (60%)			117 (41%)	152 (53%)	
Secondary (9-12)	114 (39%)	108 (37%)			153 (53%)	118 (41%)	
Secondary (13+)	7 (2%)	8 (3%)			18 (6%)	16 (6%)	
**Wealth index:**				0.528			0.041
Low	184 (63%)	193 (67%)			109 (38%)	138 (48%)	
Middle	86 (29%)	77 (27%)			129 (45%)	113 (39%)	
High	23 (8%)	18 (6%)			50 (17%)	37 (13%)	
**Travel time to health facility:**				0.016			0.689
0-29 min	71 (24%)	85 (30%)			72 (27%)	79 (28%)	
30-60 min	140 (48%)	104 (36%)			100 (37%)	98 (35%)	
61-120 min	51 (17%)	72 (25%)			61 (22%)	71 (25%)	
Over 121+ min	31 (11%)	27 (9%)			38 (14%)	32 (11%)	
**Household phone ownership:**				0.047			0.001
No	121 (41%)	96 (33%)			13 (5%)	39 (14%)	
Yes	172 (59%)	192 (67%)			275 (95%)	249 (86%)	
**Woman’s personal phone ownership:**				<0.001			0.136
No	46 (27%)	10 (5%)			34 (13%)	42 (17%)	
Yes	125 (73%)	182 (95%)			237 (87%)	202 (83%)	

*All data are unweighted.

### Mobile phone ownership

At baseline, more control district households owned a mobile phone. Mobile phone ownership increased in both intervention and control areas during the study period and was higher in the intervention district at endline. Of households with phones, significantly more women in the control district at baseline said they owned their own phone than women in the intervention district, but there were no differences in the final survey (**Table 1**).

### Enrollment in Liga Inan

**Table 2** shows that 70% of all surveyed women in the intervention district participated in Liga Inan. Rates remained high across all sub-districts with the most rural and remote of four sub-districts (Turiscai) having the lowest enrollment (60%). Women eligible to participate in Liga Inan, determined by their report of owning a mobile phone and attending at least one ANC visit where women could be enrolled, had a 77% participation rate. Among women enrolled in Liga Inan, 93% reported receiving messages at least once a week (data not shown). Enrollment varied by wealth index: among surveyed women in the highest wealth category, 79% participated in Liga Inan, compared to 69% of medium wealth rank and 58% in the lowest wealth category (data not shown).

**Table 2:** Percent enrollment in Liga Inan in the intervention sub-districts for all surveyed women and for those eligible to enroll

Manufahi district	% enrollment of all surveyed women (n = 288)	% enrollment of eligible* surveyed women (n = 186)
Alas	68%	71%
Fatuberliu	79%	81%
Same	71%	78%
Turiscai	60%	70%
**Manfuahi District Total**	**70%**	**77%**

*Owned a mobile phone and had at least one antenatal care visit.

### Utilization of maternal health services

#### Antenatal care (ANC) visits

Rates of women having four or more ANC visits did not differ significantly between districts at baseline or final surveys (Table 3). Multivariate analysis did not show a significant association of the Liga Inan intervention with receipt of four or more ANC visits (odds ratio OR = 1.0; 95% confidence interval CI = 0.54-1.9; *P* < 0.999).

**Table 3:** Bivariate and multivariate results for key indicators from baseline and final surveys in intervention and control districts with significance of differences

Variable	Baseline			Final			Multivariate analysis	
**Intervention N = 293**	**Control N = 288**	***P*-value**	**Intervention N = 288**	**Control N = 288**	***P*-value**	**Odds ratio**	***P*-value**
% 4 or more ANC visits	76%	67%	0.159	85%	81%	0.451	1.0* (0.54-0.9)	0.999
% skilled birth attendant	48%	38%	0.153	62%	36%	0.000	1.9* (1.1-3.2)	0.022
% deliver in health facility	32%	29%	0.645	49%	28%	0.002	1.9* (1.1-3.6)	0.023
% postpartum visit in 2 days	26%	38%	0.069	51%	25%	0.000	4.7† (2.4-9.0)	0.001
% newborns postnatal visit within 2 days	20%	32%	0.053	39%	22%	0.009	5.5† (2.9-10.4)	0.001

ANC – antenatal care

*Adjusted for age, education, distance to a health facility, and wealth.

†Adjusted for age, education, distance to health facility, wealth and use of skilled birth attendant.

#### Skilled birth attendance (SBA) and facility delivery

District-level rates of SBA and facility delivery were similar at baseline, but in the final survey, the rates of both SBA and facility delivery were significantly higher in the intervention district (**Table 3**). Multivariate analysis showed that women in the intervention district had nearly twice the odds of using an SBA (OR = 1.9; 95% CI = 1.1-3.2; *P* < 0.022) and having a facility delivery (OR = 1.9; 95% CI = 1.1-3.6; *P* < 0.023) compared to women at baseline and in the control district.

The final survey showed that among Liga Inan participants who reported they had contacted health staff through the system, 78% had an SBA and 51% a facility delivery. Among enrolled women who did not contact health staff while pregnant, rates of both SBA and facility delivery were substantially lower, 41% and 24% respectively (data not shown).

#### Postpartum and postnatal care visits within two days

District-level baseline rates for both postpartum care (PPC) within two days of delivery and for newborn care were similar, but in the final survey, both were significantly higher in the intervention district. Controlling in multivariate analysis for use of a skilled birth attendant as well as age, education, distance to a health facility, and wealth, women in the intervention district had nearly five times the odds of having PPC within two days of delivery compared to women at baseline and in the control district (OR = 4.7; 95% CI = 2.4-9.0; *P* < 0.001). Newborns in the intervention district had over five times the odds of receiving newborn care within two days of delivery compared to baseline and control rates (OR = 5.5; 95% CI = 2.9-10.4; *P* < 0.001).

## DISCUSSION

This study supports the potential value of integrating mHealth programs into national systems of maternal and newborn health services. For pregnant women in the district with the mobile phone intervention that was part of routine maternal care, the likelihood of having an SBA and a health facility delivery doubled when compared to baseline and control. Women in the intervention district also had nearly five times the odds of having a postpartum check and over five times the odds of having their newborns checked within two days of delivery. These increased rates of service utilization were not isolated to one or two areas but were noted across all four sub-districts in the intervention district, in both peri-urban and rural areas. The findings are consistent with other studies of mobile phone use to improve utilization of maternal health services [21-25].

A key element of this program was integration of the mHealth services within regular MoH care in a setting with high rates of both ANC attendance and mobile phone ownership. Although the exact mechanisms through which Liga Inan may have influenced participants is not known, bridging a communication gap between pregnant women and health staff appeared to be an important element of its success. Jareethum et al found that the provision of text messaging via mobile phones resulted in pregnant women experiencing increased confidence and decreased anxiety levels during the antenatal period [26]. In Liga Inan, regular automated messages appeared to enhance the perceived relationship between mothers and their midwife or other health care provider; mothers sometimes sent replies to a text saying “thank you” after receiving the automated messages.

In addition to twice weekly text messages encouraging healthy behaviors, the Liga Inan system supports mothers to contact their health provider directly through voice communication, and women’s use of that system was associated with higher rates of SBA and health facility deliveries than for those who did not use it. It may be that improved communication via Liga Inan led to higher use of the health care system for deliveries, which in turn resulted in the greater use of postpartum and newborn care services. The MoH’s own independent evaluation of Liga Inan, completed in 2016, agreed that the program facilitated communication and connections between midwives and their patients [17].

Increasing rates of ANC is sometimes suggested as a pathway to increasing skilled birth attendance. Although the rate of attendance at four or more ANC visits increased in the control district from 67% at baseline to 81% in the final survey, there were no increases in either SBA or facility delivery in that district during the study period. This finding suggests that provision of ANC alone, even the recommended four visits, may not be sufficient to produce changes in the use of health care for deliveries.

Enrollment in the Liga Inan program, however, was itself associated with higher rates of maternal services use. A separate unpublished analysis in the intervention district found that 67% of Liga Inan-enrolled women reported having an SBA compared to 35% of unenrolled women. Similarly, 42% of participating women delivered in a health facility compared to 22% of women not participating in Liga Inan [27].

The integration of Liga Inan into routine ANC visits created a strong sense of program ownership among MoH health staff, many of whom became its champions. A separate qualitative study exploring the impact of Liga Inan on midwives’ workload and job satisfaction found that they enthusiastically supported the program. Midwives stated that it helped them work more efficiently, assisted them to monitor the condition of their pregnant clients, and provided them with a tool to achieve MoH mandates to increase utilization of maternal health services [28]. The Liga Inan evaluation conducted by the MoH recommended that the program should be incorporated into the national systems for maternal and child health and for health promotion [17].

Baseline data confirmed a relatively high rate of mobile phone ownership in both intervention and control districts, rates that increased substantially during the study period. One common concern about mHealth programs is mobile phone ownership may be limited for women. In our study, the vast majority of women reported having their own phone, assuring that when text messages arrive they are delivered to the intended recipient. Another common concern is fear that poorer families will be less likely to have mobile phones, and thus less able to benefit from an mHealth program [29]. In our study, although phone ownership varied by level of wealth, including the wealth index in the multivariate analysis did not reduce the odds of the intervention effect.

This study has the expected limitations of a program-focused evaluation. It uses a quasi-experimental rather than experimental design, which limits the strength of the findings. The study design controlled for several potential confounding characteristics between the intervention and the control districts, however, not all characteristics can be controlled statistically, so differences found after the intervention may be due to factors inherent in the districts rather than the intervention itself. Similar spurious findings could result from the passage of time, and unobserved intervening events may have differed between the two districts.

## CONCLUSIONS

Our findings support other mHealth research demonstrating that mobile phones can be successful behavior change tools to increase appropriate use of maternal health services. In addition, the Liga Inan results suggest that mHealth can be effective when integrated into routine MoH services in a manner that is potentially scalable and sustainable. As of 2019, Liga Inan is active in all 13 of Timor-Leste’s districts, with the MoH gradually assuming management and financial responsibility for the program.
